# Traumatic bilateral carotid artery dissection following severe blunt trauma: a case report on the difficulties in diagnosis and therapy of an often overlooked life-threatening injury

**DOI:** 10.1186/s40001-015-0153-1

**Published:** 2015-07-22

**Authors:** Moritz Crönlein, Gunther H Sandmann, Marc Beirer, Silke Wunderlich, Peter Biberthaler, Stefan Huber-Wagner

**Affiliations:** Department of Trauma Surgery, Klinikum rechts der Isar, Technical University Munich, Ismaninger Strasse 22, 81675 Munich, Germany; Department of Neurology, Klinikum rechts der Isar, Technical University Munich, Ismaninger Strasse 22, 81675 Munich, Germany

**Keywords:** Neck injuries, Traumatic vessel dissection, Internal carotid artery dissection, Polytrauma

## Abstract

**Background:**

Traumatic carotid artery dissections are very rare, often overlooked and life-threatening injuries. Diagnosis and treatment are difficult especially in multiple injured patients.

**Case presentation:**

We report on a 28-year-old female major trauma patient (injury severity score, ISS 50) who was involved in a motor vehicle accident. She was primarily transferred to a level II trauma center. After initial assessment and operative management, an anisocoria was diagnosed on the intensive care unit. Subsequent CT angiography and extracranial duplex sonography revealed a bilateral internal carotid artery dissection. The patient was transferred to our level I trauma center where conservative treatment with high-dose heparin therapy was started at day two after trauma. Outcome after 6 months was very good.

**Conclusion:**

Besides presenting the case and outcome of this patient, the article discusses the diagnostic and therapeutic management of this extremely rare and often overlooked dangerous injury. To avoid overlooking carotid artery dissections, CT angiography of the neck region should be generously included into the initial multislice CT whole-body scan, when the injury results from an according trauma. For the best outcome, sites of hemorrhage should be abolished quickly and the anticoagulative therapy should be initiated as soon as possible. Interdisciplinary treatment of trauma surgeons and neurologists is crucial.

## Background

Traumatic carotid artery dissections are often overlooked life-threatening injuries [1]. Even though most of the carotid artery dissections occur spontaneously, about 4% of the dissections are related to severe trauma [2]. While spontaneous dissections are usually seen in older patients, traumatic dissections mostly affect young patients [3]. The diagnosis of traumatic dissections is often difficult, because of missing initial symptoms or distracting injuries. The treatment itself also provides many difficulties, especially when combined with other injuries after severe trauma [2]. Therefore, improvements in diagnosis and treatment of traumatic artery dissections are of relevance for emergency medicine.

## Case presentation

A 28-year-old woman was involved in a motor vehicle accident (MVA) and suffered major trauma. The trauma mechanism was a high speed MVA with head-on collision on a motorway. When the paramedics arrived, the patient was found lying on the street next to the car involved in the accident, she was soporific with an initial Glasgow Coma Scale (GCS) of 8. Furthermore, she complained about severe pain in her head, chest and right leg. She was intubated, immobilized and transferred by ground emergency to a Level II trauma center nearby.

**Figure 1:**
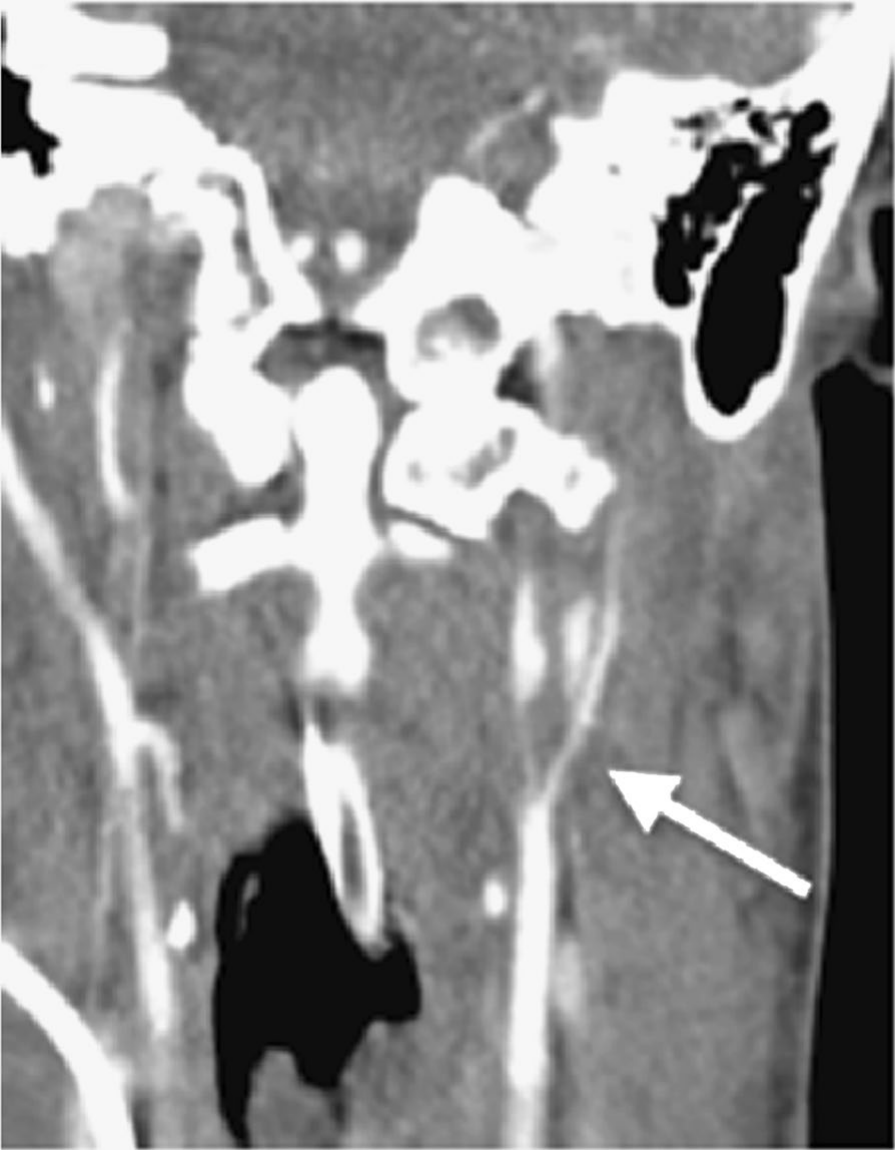
Second CT scan after ICU admission with coronal reconstructions of the bilateral ICA dissection. “String sign” indicated with *an arrow*.

After initial trauma management following the Advanced Trauma Life Support (ATLS)-scheme, a whole-body CT scan was performed. As direct sequelae of trauma, multiple injuries were found (see Table 1), but no traumatic brain injury (ISS = 50 points). Following the damage control concept, the II° open tibial fracture was stabilized with an external fixator, followed by surgery of the open mandibular fracture. The patient was transferred to the intensive care unit (ICU) after the operation. The postoperative checkup on the ICU 4 h after hospital admission showed an anisocoria (right > left). Therefore, additional diagnostics were initiated. A CT angiography revealed the suspicion of a bilateral internal carotid artery (ICA) dissection (Figure 1). The suspected diagnosis was confirmed by subsequent extracranial duplex sonography.

**Table 1:** Pattern of injuries of the patient presented

Pattern of injuries resulting in an ISS of 50 points	AIS score
Bilateral carotid artery dissection with bilateral stroke	5
Open mandibular fracture	4
Le Fort I fracture	2
Undislocated fracture of the left clavicula	1
Rib fracture of the first rib	1
Bilateral lung contusion	2
Pneumothorax	3
Pelvic ring fracture type A	2
II° open tibial head fracture	2

48 h after the accident, the patient was transferred via helicopter non-intubated to the emergency department of our level I trauma center. It was decided not to intubate the patient prior helicopter transport to make continuous neurological assessment possible. The patient presented with a GCS of 8 under sufentanil analgesia, in soporific condition, not focusing with her eyes, spontaneous movements of the right arm, her left leg moved upon stimulation and the left arm and right leg were not moved spontaneously but after stimulation, no speech production, with persisting anisocoria (right > left) in terms of an incomplete horner syndrome. The additional CT angiography showed no changes in the vascular findings but a cerebral infarction in the left central region.

**Figure 2:**
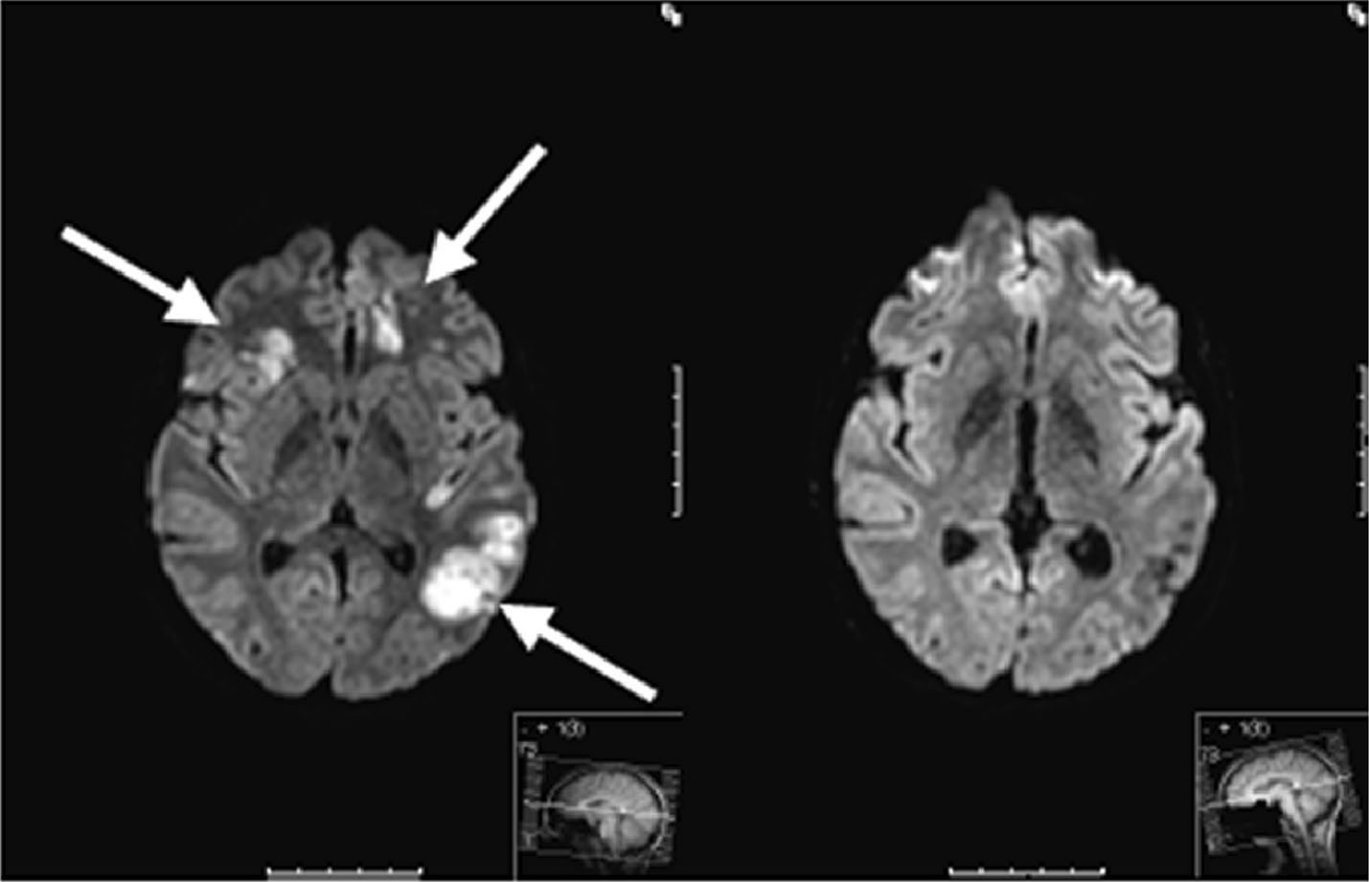
Axial T-2 sequences of a brain MRI scan of the 28-year-old patient. *Left* posttraumatic bihemispheric, mainly left-sided, ischemic lesions near the border zone, 3 days after trauma (indicated with *arrows*). *Right* 6 months follow-up MRI scan with good recovery.

The patient was transferred to the ICU. A transcranial Doppler sonography showed a sufficient collateral circulation of the middle cerebral artery via the posterior communicating arteries, so that an interventional stent angioplasty (with subsequent need of double platelet aggregation inhibition) was not performed. High-dose intravenous heparinization was started immediately (PTT 60–80 s). Magnet Resonance Imaging (MRI) on the next day (3 days after trauma) (Figure 2) revealed bihemispheric, mainly left-sided, ischemic lesions near the border zone. The high-dose heparinization was continued.

**Figure 3:**
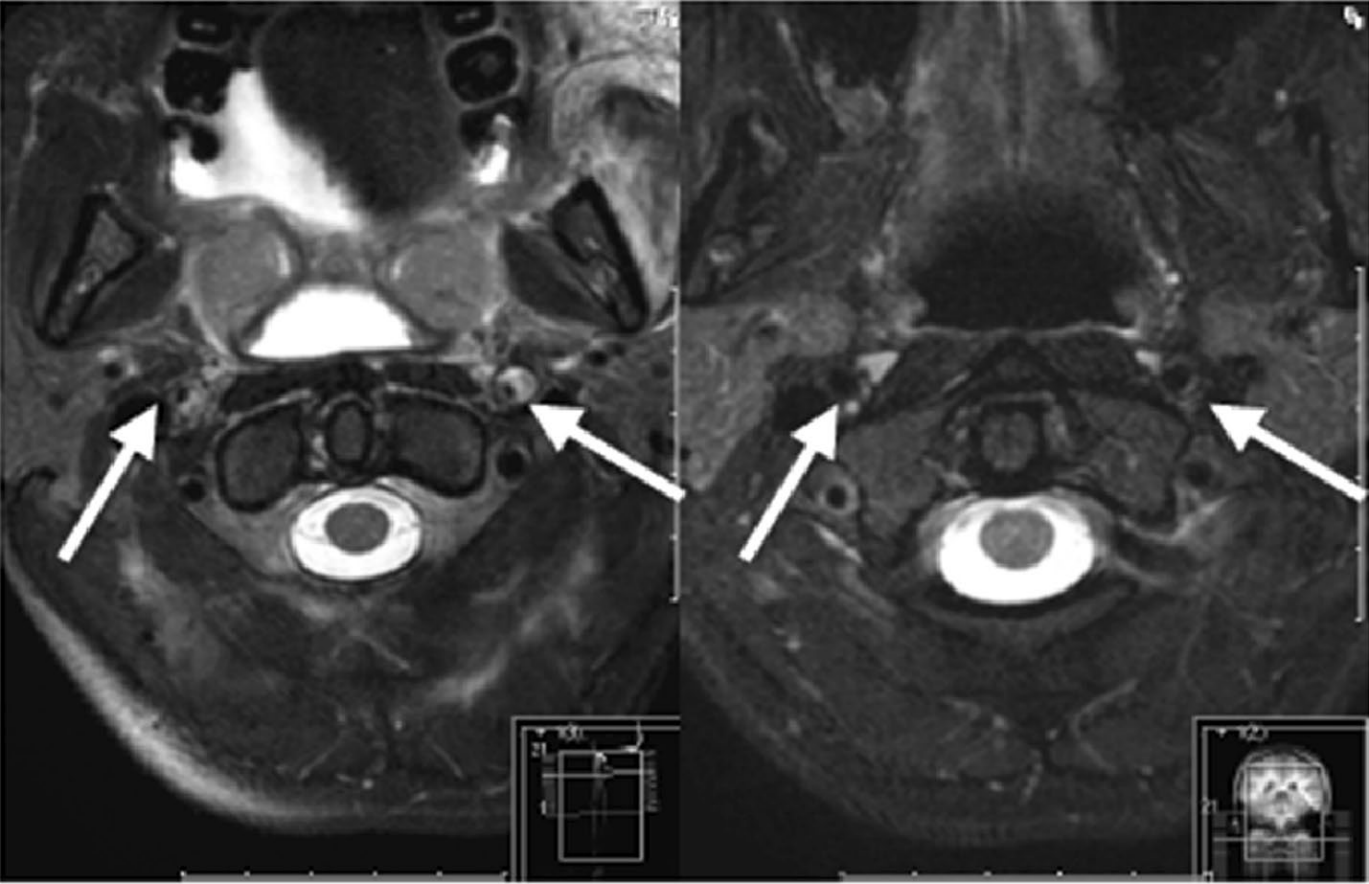
Axial T-2 sequences of a brain MRI Angio scan of the 28-year-old patient. *Left* posttraumatic intracranial dissection with intramural hematomas of both internal carotid arteries 3 days after trauma (indicated with *arrows*). *Right* 6 months follow-up MRI scan with good perfusion of both internal carotid arteries.

Eight days after the accident and ICU treatment the patient was referred to the stroke unit with a score of 8 on the National Institutes of Health Stroke Scale (NIHSS, min. 0 points = no neurological deficit, max. 42 points = worst outcome). Over the course of time the proximal tibial fracture was treated with osteosynthesis and the intravenous heparinization was converted to oral anticoagulation with Dabigatran (Pradaxa®) 150 mg 1–0–1 (off-label use because of needle-phobia). The clinical situation improved, but regarding focal neurological deficiencies, a global motoric aphasia remained. The patient was discharged to rehabilitation 22 days after the trauma with a NIHSS of four points. Six months later, the patient presented at our clinic for follow-up examinations and showed great recovery. Besides slight concentration difficulties and fine motor dysfunctions, no neurological deficiencies remained (NIHSS = 0). The MRI scan 6 months after trauma showed good recovery with a good perfusion of both internal carotid arteries (Figures 1, 2, 3).

## Discussion

### Incidence

Most of the carotid artery dissections (CADs) occur spontaneously [2]. Only about 4% of all CADs result from a (poly-) trauma such as a motor vehicle accident (MVA) [4]. Bilateral ICA dissection following blunt trauma, as described in our case, have a special role in the current literature, because they are only very rarely described. Exact data on the incidence of traumatic CAD do not exist. Bilateral traumatic carotid artery dissection is extremely rare.

### Pathophysiology

Different injury mechanisms for traumatic CADs are described in the current literature. Distraction/extension, distraction/flexion or lateral flexion forces of the C-spine may result in traumatic ICA dissections [2, 5]. Even a vasocompression between C-Spine and mandibula during a hyperinclination trauma can lead to a dissection of the internal carotid artery (ICA) [1, 2]. These mechanisms are frequently seen in MVAs, where the car occupants remain fixed in their seats by the security belt during a sudden loss of speed (i.e. crash, full braking, etc.). The mentioned forces may cause small lesions of the vessel wall, which can result in intimal tears, intramural hematomas or complete lumen displacement [2, 4]. It is reasonable for cerebral ischemic insults to activate the coagulation cascade, which results in thrombus formation and, concomitantly, in an increased risk of thromboembolic cerebral insults [1, 2, 6].

Traumatic dissections of the arteries in the head and neck region are most frequently located in the mobile segments of the vessels. Concerning the ICA most of the traumatic dissection occur below the scull base [6–8].

### Diagnosis

There are several options to diagnose vessel dissections used in the clinical routine. Duplex ultrasound is one of the non-invasive methods with a high disposability even in smaller hospitals. It is a common method to detect cerebrovascular injuries [1], although it has certain limits when it comes to acute traumatic dissections in major injuries: it is an operator-dependent method with poor vision of the intracranial aspects of a dissection and it also gives limited information about small intimal tears [6]. When it comes to dissections of the ICA as described in our case, the sensitivity of diagnostic ultrasound is even lower [9, 10]. According to the given limits, diagnostic ultrasound is rather recommended as an additional method especially to evaluate the progress of a dissection [2, 6, 11].

The MRI scan combined with the MR angiography is considered to be the gold standard for detection of carotid artery dissections [2, 12]. It determines dissections in up to 99% and provides additional information about concomitant injuries (i.e. brain injuries, fractures, etc.) [1, 6, 13, 14]. But it also has certain limits, especially during the diagnostic of critically injured patients in the trauma room. Besides the long duration of the scan, there are on the one hand restrictions in the use of potentially life supporting devices (i.e. pacemakers/iron-based metal implants, etc.) and on the other hand there is the lack of availability of MRI scanners especially in the smaller trauma centers [5].

The CT scan combined with CT angiography are prominently used as diagnostic features for the detection of cerebrovascular injuries, especially when thinking of the acute situation in the emergency room of severely injured patients. “String signs”, constrictions of the lumen, are reckoned to be indirect signs of artery dissection (Figure 1) [6]. Although it is not recommended by the AWMF (“Arbeitsgemeinschaft der Wissenschaftlichen Medizinischen Fachgesellschaften”) guidelines as standard method yet [1, 9], the further development of the CT scanners (>64 slices) shows comparable results in sensitivity to the MRI/MRA scans [2, 15]. The use of whole-body CT scans in severely injured patients was described by Huber-Wagner et al. [16]. However, to get additional information about a possible cerebrovascular injury, it can be necessary to include CT angiography into the protocol of the whole-body CT. Wick et al. showed how to avoid the under diagnosis of blunt cervical vascular injuries with the use of CT angiography included in the multislice CT (MS-CT) in their “Innsbruck Emergency Algorithm” [17]. To rule out the patients that need an additional CT angiography the modified “Denver Screening Criteria” (Table 2) can be helpful [2]. CT angiography should be performed generously in severely injured patients following the above-mentioned criteria [18].

**Table 2:** Modified Denver screening criteria for blunt cerebrovascular injuries [2]

Signs of blunt cerebrovasuclar injury	Risk factors for blunt cerebrovascular injury
Arterial bleeding	High-energy trauma mechanism combined with: Le Fort II/III fracture Cervical spine fractures (subluxations, CI-CIII fractures, fractures extending to the transverse foramen) Basilar skull fracture with carotid canal involvement Diffuse axonal injury with GCS ≤6 Near hanging with anoxic brain injury Combination of traumatic brain injury and thorax trauma
Cervical hematoma	
Focal neurological deficiency	
Neurological findings not matching with CT findings	
Ischemic insult seen on a secondary CT scan	
Cervical heart murmur	

### Therapeutical options

To treat cerebrovascular dissections, there are several therapeutical options including open surgery (microsurgical vessel sutures, extracranial/intracranial bypass, thromboendarterectomy), endovascular management (stenting, stent-assisted endovascular thrombolysis, stent-assisted thrombectomy), thrombolysis and antithrombotic [2, 6].

The German Society of Neurology (DGN) gives the following advice for the treatment of spontaneous cerebrovascular dissections in the acute phase, guidelines for traumatic dissections do not exist so far [1, 9, 15]:

- thrombolysis is possible in patients with cerebral ischemia following dissection within 4.5 h after initial symptoms
- endovascular treatment, including intraarterial thrombolysis, in the acute phase is only justified in rare cases
- hypertension and intensive care monitoring can be useful if severe cerebral perfusion disorders without any embolic occlusions occur.

Surgical options as mentioned above are difficult and risky in the acute phase of the dissection [2, 19]. This is why they usually do not belong to the first line therapy in the acute phase. However, there are certain cases where operative treatment is needed (i.e. to restore the blood flow in a case of distinct tear of the vessels or complete occlusions without ischemic intracranial lesions) [1, 8].

Endovascular treatment of traumatic cervical ICA injuries is becoming more and more popular and is meant to be an attractive minimally invasive alternative to surgery [19]. In the current literature, endovascular treatment is described to be a good therapeutic option even for traumatic dissections [1, 20, 21]. But there are still certain risk factors including stroke, perforation of the thin dissected arteries, etc., that have to be considered [19]. Further randomized trials with a high level of incidence are still needed [1, 6].

Systemic and local lysis are thought to be useful especially to prevent from thromboembolic cerebral insults, but still present high risks in the setting of a severely injured patient with multiple fractures (as presented in our case) [2, 6, 9].

In our case of a severely injured patient with bilateral ICA dissections, treatment has to be determined interdisciplinary. To prevent the patient from further thromboembolic events, a therapeutic anticoagulation is considered to be the treatment of choice [1, 2, 9, 19, 22]. In general, the earliest possible time to start with the anticoagulation is best. If there are other injuries with high risk of severe bleeding (i.e. pelvic fractures), the time to start with the anticoagulation should be considered in an interdisciplinary approach (trauma surgeons, orthopedic surgeons, neurologists, neurosurgeons and radiologists). The ATLS principle “treat first what kills first” should be supplied at any time of the treatment. According to the guidelines, the therapeutic intravenous heparinization with a PTT of 50–70 s followed by oral anticoagulation with warfarin or phenprocoumon (INR 2–3) for at least 3 months should be warranted [2, 6].

## Conclusion

Approximately 4% of the CADs result from trauma, such as MVAs. Bilateral traumatic carotid artery dissections as presented in the case at hand are extremely rare. There are several therapeutical options to treat patients with traumatic dissections of the ICA. But when it comes to a severely injured patient as described in our case, no specific guidelines for diagnosis and treatment exist. To avoid overlooking a carotid artery dissection, we recommend to include a CT angiography of the neck region into the MS-CT whole-body scan generously, when a certain trauma mechanism shows the possible coexistence of an artery dissection. Regarding the therapeutical options, the bleeding risk is one of the most challenging aspects. Therefore, a close cooperation between the different medical disciplines is essential. Following the ATLS principle “treat first what kills first”, the patient should be stabilized initially, sites of hemorrhage should be abolished quickly (i.e. fracture stabilization with external fixator, etc.) and the anticoagulative therapy should be initiated as soon as possible.

## Consent

Written informed consent was obtained from the patient for publication of this case report and any accompanying images. A copy of the written consent is available for review by the editor of this journal.

## Abbreviations

AIS: Abbreviated Injury Scale
ATLS: Advanced Trauma Life Support
CAD: carotid artery dissection
GCS: Glasgow Coma Scale
ICA: internal carotid artery
ISS: Injury Severity Scale
MCA: middle cerebral artery
MVA: motor vehicle accident
NIHSS: National Institute of Health Stroke Scale

## References

[1] Lenz M, Bula-Sternberg J, Koch T, Bula P, Bonnaire F (2012). Traumatic dissection of the internal carotid artery following whiplash injury. Diagnostic workup and therapy of an often overlooked but potentially dangerous additional vascular lesion. Der Unfallchirurg.

[2] Jansen G, Popp J, Dietrich U, Mertzlufft F, Bach F (2013). Traumatic dissection of the carotid artery: challenges for diagnostics and therapy illustrated by a case example. Der Anaesth.

[3] Lleva P, Ahluwalia BS, Marks S, Sahni R, Tenner M, Risucci DA (2012). Traumatic and spontaneous carotid and vertebral artery dissection in a level 1 trauma center. J Clin Neurosci Off J Neurosurg Soc Australas.

[4] Marschner-Preuth N, Warnecke T, Niederstadt TU, Dittrich R, Schabitz WR (2013). Juvenile stroke: cervical artery dissection in a patient after a polytrauma. Case Rep Neurol.

[5] Inamasu J, Guiot BH (2006). Vertebral artery injury after blunt cervical trauma: an update. Surg Neurol.

[6] Mohan IV (2014). Current optimal assessment and management of carotid and vertebral spontaneous and traumatic dissection. Angiology.

[7] Downer J, Nadarajah M, Briggs E, Wrigley P, McAuliffe W (2014). The location of origin of spontaneous extracranial internal carotid artery dissection is adjacent to the skull base. J Med Imaging Radiat Oncol.

[8] Okada Y, Shima T, Nishida M, Yamane K, Kagawa R (1995). Traumatic dissection of the common carotid artery after blunt injury to the neck.pdf. Surg Neurol.

[9] Fachgesellschaften (2008) AAdWM: Leitlinie Dissektionen hirnversorgender Arterien.pdf. AWMF online

[10] Baumgartner RW, Arnold M, Baumgartner I, Mosso M, Gonner F, Studer A (2001). Carotid dissection with and without ischemic events: local symptoms and cerebral artery findings. Neurology.

[11] Tahmasebpour HR, Buckley AR, Cooperberg PL, Fix CH (2005) Sonographic examination of the carotid arteries. Radiographics 25:1561–1575 10.1148/rg.256045013 16284135

[12] Vertinsky AT, Schwartz NE, Fischbein NJ, Rosenberg J, Albers GW, Zaharchuk G (2008). Comparison of multidetector CT angiography and MR imaging of cervical artery dissection. AJNR Am J Neuroradiol.

[13] Patel RR, Adam R, Maldjian C, Lincoln CM, Yuen A, Arneja A (2012). Cervical carotid artery dissection: current review of diagnosis and treatment. Cardiol Rev.

[14] Phan T, Huston J, Bernstein MA, Riederer SJ, Brown RD (2001). Contrast-enhanced magnetic resonance angiography of the cervical vessels: experience with 422 patients. Stroke.

[15] Neurologie (2012) DDGf: Spontane Dissektion der extrakraniellen und intrakraniellen hirnversorgenden Arterien. Leitlinien für Diagnostik und Therapie in der Neurologie

[16] Huber-Wagner S, Biberthaler P, Haberle S, Wierer M, Dobritz M, Rummeny E (2013). Whole-body CT in haemodynamically unstable severely injured patients: a retrospective, multicentre study. PLoS One.

[17] Wick MC, Weiss RJ, Lill M, Jaschke W, Rieger M (2010). The “Innsbruck Emergency Algorithm” avoids the underdiagnosis of blunt cervical vascular injuries. Arch Orthop Trauma Surg.

[18] Berne JD, Cook A, Rowe SA, Norwood SH (2010). A multivariate logistic regression analysis of risk factors for blunt cerebrovascular injury. J Vasc Surg.

[19] Rao AS, Makaroun MS, Marone LK, Cho JS, Rhee R, Chaer RA (2011). Long-term outcomes of internal carotid artery dissection. J Vasc Surg.

[20] Seth R, Obuchowski AM, Zoarski GH (2013). Endovascular repair of traumatic cervical internal carotid artery injuries: a safe and effective treatment option. AJNR Am J Neuroradiol.

[21] Jindal G, Fortes M, Miller T, Scalea T, Gandhi D (2013). Endovascular stent repair of traumatic cervical internal carotid artery injuries. J Trauma Acute Care Surg.

[22] Norris JW (2005). Extracranial arterial dissection: anticoagulation is the treatment of choice: for. Stroke.

